# The mean severity score and its correlation with common computed tomography chest manifestations in Egyptian patients with COVID-2019 pneumonia

**DOI:** 10.1186/s43055-020-00368-y

**Published:** 2020-12-08

**Authors:** Mona A. F. Hafez

**Affiliations:** Radiology and Intervention Department, Kasralainy Hospital, Cairo University School of Medicine, Kasr Al-Aini Street, Cairo, 11562 Egypt

**Keywords:** Computed tomography, COVID-19 pneumonia, Chest, Egyptian

## Abstract

**Background:**

Computed tomography (CT) is one of the main diagnostic tools for early detection and management of coronavirus disease 2019 (COVID-19) pneumonia. This study aims to highlight the commonly encountered CT findings in patients with COVID-19 pneumonia in Egypt and the mean severity score and its correlation with the imaging findings. This study involved 200 patients with pathologically confirmed COVID-19 infection; non-contrast CT chest was performed for all cases; in addition, CT findings and severity score (CT-SS) were then assessed using descriptive analysis, and the correlation between the CT findings and disease severity was assessed.

**Results:**

The ground-glass densities and peripheral adhesions were the most typical CT findings. Prominent interlobular septations; bronchial thickening/dilatation; CT signs of crazy-paving, halo, and reversed halo; and reactive mediastinal lymphadenopathy were significantly correlated with disease severity. The mean CT-SS of Egyptian patients with COVID-19 pneumonia was 11.2 (mild to moderate severity).

**Conclusion:**

Multislice CT played a vital role in the early identification of Egyptian patients with COVID-19 pneumonia. The assessment of the CT severity score of COVID-19 is essential for the extent of pneumonia involvement to help clinicians achieve the purpose of early diagnosis and accurate treatment.

## Background

Coronavirus disease 2019 (COVID-19) pneumonia is a recently discovered rapidly spreading acute respiratory syndrome [1]. Egypt is one of the top 40 countries worldwide according to the total number of cases in August 2020 [2].

During the current epidemic in Egypt, computed tomography (CT) is used as the main diagnostic tool for early detection and management of COVID-19 pneumonia. Laboratory tests were the standard for diagnosing COVID-19 pneumonia. However, sometimes, they are unavailable in an emergency, and the results are unavailable immediately [3, 4].

Chest CT has a 97% sensitivity for the diagnosis of COVID-19 pneumonia after a mean interval of 5 days [3]. The typical chest CT findings in COVID-19 pneumonia are bilateral, peripheral, and basal predominant ground-glass opacities (GGOs) with or without consolidation and bronchovascular thickening [5]. In addition, atypical chest CT findings include central upper lobe predominance, masses, nodules, cavitations, tree-in-bud sign, lymphadenopathy, and pleural effusion [6].

Quantitative and semiquantitative indicators that evaluate the chest CT severity score (CT-SS) of lung inflammation in COVID-19 assess COVID-19 burden and provide an objective approach in rapidly identifying patients in need of hospital admission. The CT-SS is an adjustment of a method previously used in patients with severe acute respiratory syndrome (SARS) to describe the extent of the disease in the lungs, which was correlated with clinical and laboratory parameters [7].

This study highlights the commonly encountered CT findings in patients with COVID-19 pneumonia in Egypt and the mean CT-SS and its correlation with the imaging findings.

## Methods

In this cross-sectional prospective study, 200 patients including 111 males (55.5%) and 89 females (44.5%) were enrolled in the period from June 30, 2020, to August 30, 2020. The male-to-female distribution was 1.2:1.

All patients clinically diagnosed or suspected with COVID-19 underwent high-resolution CT/non-contrast-enhanced multi-slice chest CT. Patients with chest CT findings suspicious of COVID-19 pneumonia were included in the study (COVID-19 Reporting and Data System [CO-RADS] 4, 5, and 6). However, normal CT chest and those with any other chronic CT chest disease as of pulmonary edema and interstitial lung disease (CO-RADS 1, 2, or 3) were excluded from the study.

All patients were subjected to *full clinical data* taking including age, sex, exposure history, clinical complaint, and laboratory parameters.

*PCR laboratory tests* were performed for all patients before or after chest CT.

All patients underwent *non-contrast-enhanced chest CT* in the radiology department using a Siemens 16-channel scope (CTAWP92544; Siemens Healthineers, Erlangen, Germany). All volumetric chest CT were assessed at lung window of 1500 WW and − 500 WL and mediastinal window of 400 WW and 60 WL using 2D coronal and sagittal planes for better assessment of the extent of the disease.

A radiologist with 15 years of experience in thoracic imaging performed the *CT image analysis*; the following parameters in each CT were assessed.

### Location and distribution of disease

The location and distribution of the disease were unilateral or bilateral; peripheral, central, or both; and upper lobe predominance, lower lobe predominance, or both.

### CT chest findings

The CT chest findings were the presence of ground-glass opacification; consolidation; special CT chest signs such as crazy-paving, halo, and reversed halo signs; spiderweb appearance; subpleural sparing; interlobular septal thickening; intralobular septal thickening; parenchymal band; subpleural band; cyst; nodule; vascular thickening; bronchial thickening; pleural thickening; pleural reaction; pleural effusion; and reactive lymph nodes (exceeding 1 cm in short-axis diameter).

### CO-RADS based on the CT findings

The level of suspicion of COVID-19 infection is graded: CO-RADS score [8]—CO-RADS 1: COVID-19 is highly unlikely, CT is normal, or there are findings indicating a non-infectious disease; CO-RADS 2: the level of suspicion of COVID-19 infection is low, and CT findings are consistent with other infections; CO-RADS 3: COVID-19 infection is unsure or indeterminate, and CT abnormalities indicate infection but are unsure whether COVID-19 is involved; CO-RADS 4: the level of suspicion is high, and most CT findings are suspicious but not extremely typical as unilateral ground-glass, confluent, or multifocal consolidations without a typical location or any other typical findings; and CO-RADS 5: the level of suspicion is high with typical CT findings.

### Semiquantitative scoring system

A *semiquantitative scoring system* was used to quantitatively estimate the pulmonary involvement of all these abnormalities based on the area involved. The CT-SS was calculated based on the extent of lobar involvement. Each of the five lung lobes was visually scored on a scale of 0–5, with 0 indicating no involvement, 1 indicating less than 5% involvement, 2 indicating 5–25% involvement, 3 indicating 26–49% involvement, 4 indicating 50–75% involvement, and 5 indicating more than 75% involvement. The total CT score was the sum of the individual lobar scores and ranged from 0 (no involvement) to 25 (maximum involvement) [9, 10].

### Statistical analysis

The Statistical Package for the Social Sciences (version 24; IBM Corp., Armonk, NY, USA) was used in the data manipulation and significance testing. Categorical data were expressed as numbers and percentages, whereas numerical data were summarized as medians. The patients were stratified into two groups according to their CT-SS: those with CT-SS of 1–17 were considered mild, whereas those with CT-SS of 18–25 were considered severe. The chi-square correlation analysis was conducted, and *p* values of < 0.1 were used to denote statistical significance. The research protocol was approved by the Institutional Human Research Ethics Committee and conducted in accordance with the Declaration of Helsinki. A written informed consent was taken from all patients who participated in the study.

## Results

### Demographic, severity, and clinical characteristics

This study involved 200 patients, including 111 males (55.5%) and 89 females (44.5%), with their ages ranging from 20 to 87 years (average age, 52.6 years).

The mild group (CT-SS of 1–17) consisted of 180 patients, whereas the severe group (CT-SS of 18–25) was composed of 20 patients.

The most affected age group was the 51–75-year age group (99 patients; 49.5%) followed by the 26–50-year age group (90 patients; 45%), then the < 25-year age group (six patients; 3%), and the > 75-year age group (five patients; 2.5%). Disease severity is significantly correlated with the 51–75 year age group (*p* = 0.033).

The most presenting symptoms are displayed in Table 1. Disease severity was significantly correlated with lower respiratory symptoms, in particular, cough, and the presence of chronic diseases.

**Table 1:** The common presenting symptoms, number of cases, and significance of severity with some significance in cases with no complain

Clinical complain	Number and percentages of cases	*p* value
No complain	25 (12.5%)	0.075*
Upper respiratory symptom (sore throat)	22 (11%)	0.175
Lower respiratory symptoms (cough, dyspnea, tachypnea, chest pain)	132 (66%)	0.059*/0.108/0.580/0.450
Fever	139 (69.5%)	0.113
Fatigue	34 (17%)	0.856
Gastrointestinal symptoms (abdominal pain, diarrhea)	5 (2.5%)	0.580/0.501
Chronic disease	51 (25.5%)	0.035*

*p value < 0.1: significant

### CT imaging findings

Chest CT findings were assessed and analyzed for all examined patients.

**Fig. 1:**
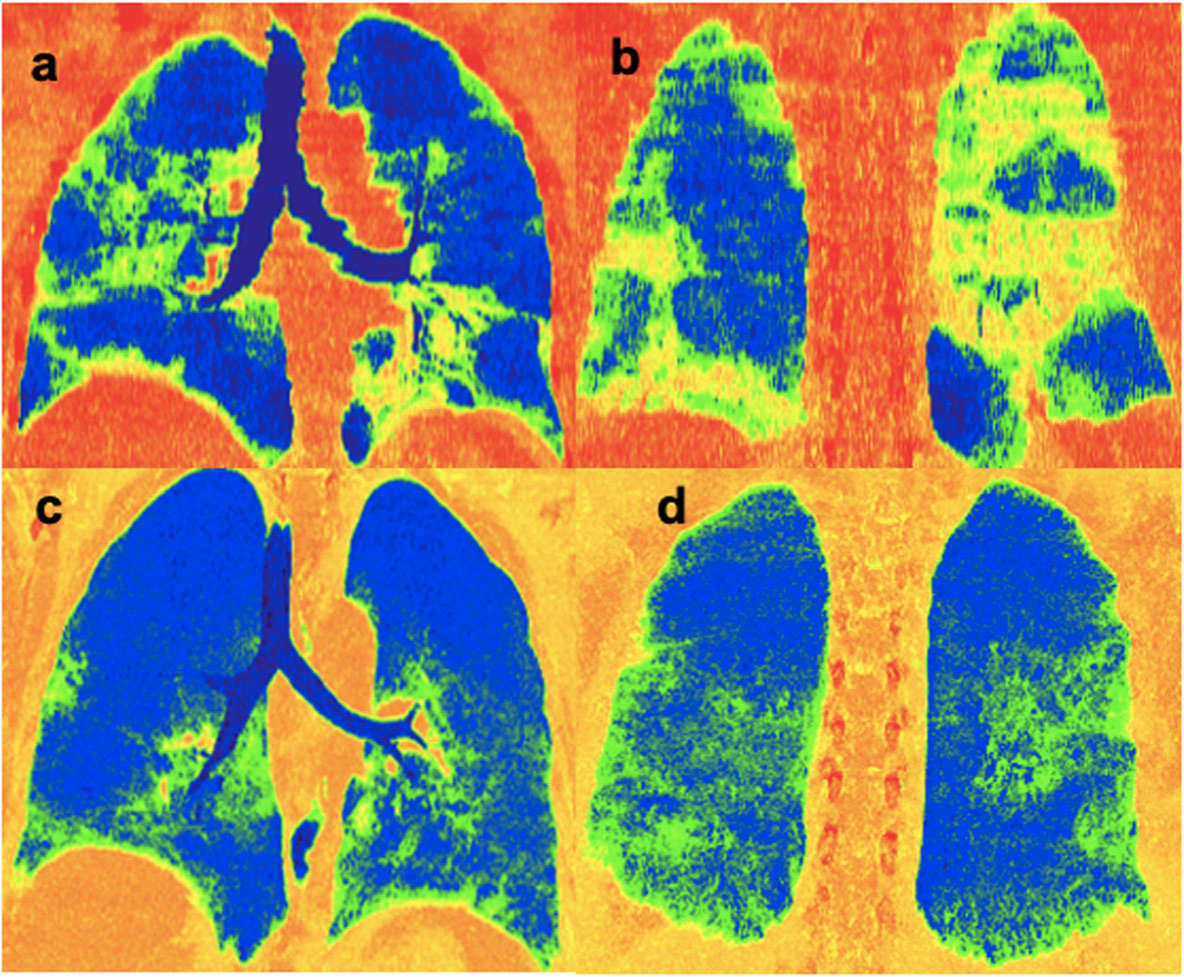
Colored spectrum two-dimensional coronal lung mid- and posterior thick slap (**a**, **b**) in a 44-year-old male patient complaining of fever, dyspnea, and cough. The patient was classified as CO-RADS 5 with a CT-SS of 20. It shows the asymmetric mainly peripheral multilobar distribution of the parenchymal lesions (green and yellow colors). **c**, **d** A 68-year-old female patient complaining of fever, dyspnea, and cough. The patient was classified as CO-RADS 5 with a CT-SS of 11. It shows the asymmetric mainly peripheral lower lobe distribution of parenchymal lesions (green colour)

Accordingly, the disease distribution was assessed; most of the cases presented with bilateral involvement with peripheral and lower lobe predominance (Table 2 and Fig. 1). Both peripheral and central distributions showed significance with disease severity.

**Table 2:** Number and percentages of cases according to their distribution and their significance of severity

Location	Number and percentage of cases	Significance (*p* value)
Unilateral	13 (6.5%)	0.214
Bilateral	187 (93.5%)	0.214
Peripheral	179 (89.5%)	0.026*
Central	2 (1%)	0.636
Both peripheral and central	19 (9.5%)	0.013*
More severity score for lower lobes	111 (55.5%)	0.602
More severity score for upper lobes	22 (11%)	0.366
Same severity score for upper and lower lobes	66 (33%)	0.229
More severity score for middle lobe/lingula	1 (0.5%)	0.738

*p value < 0.1: significant

**Fig. 2:**
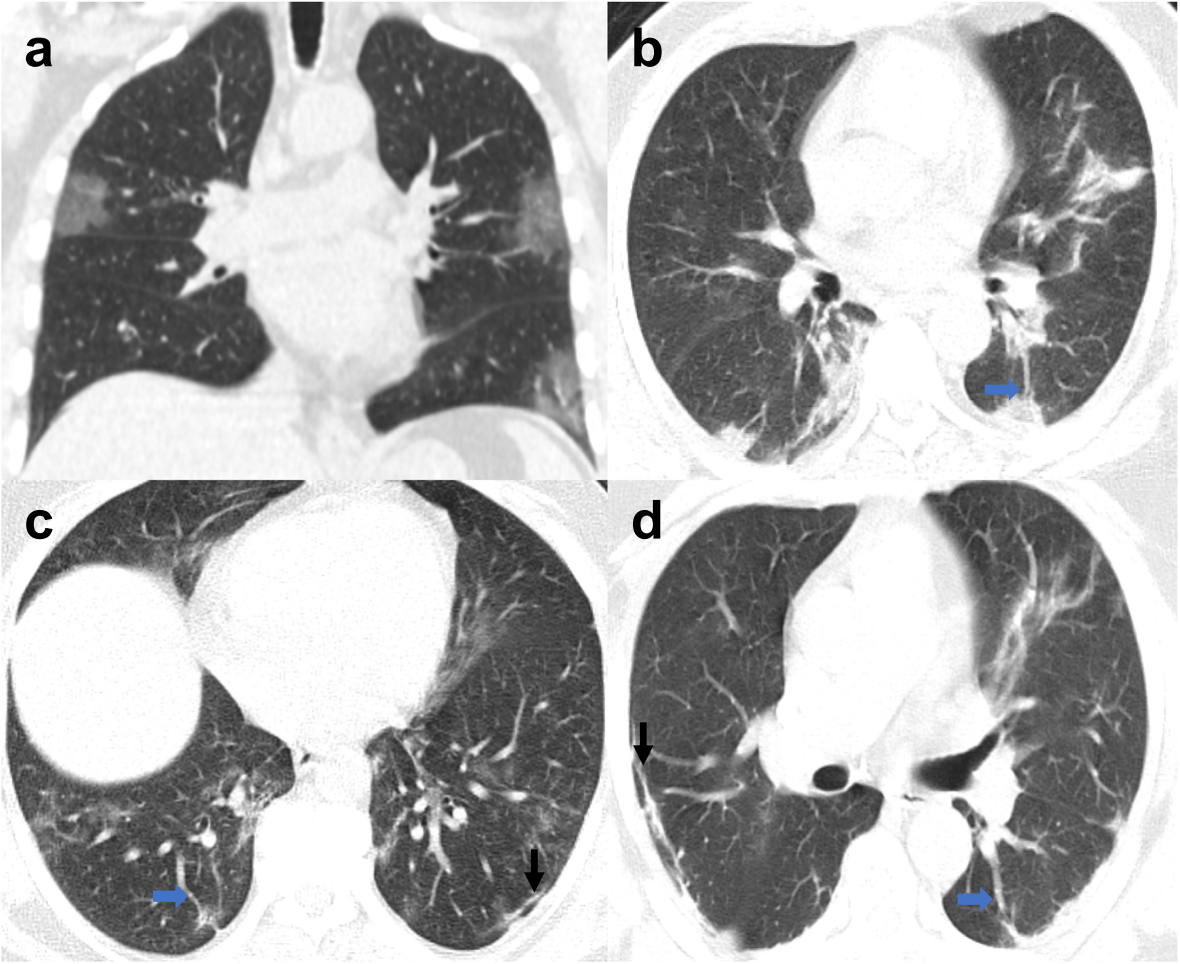
Two-dimensional coronal reconstruction (**a**) and axial CT image lung window (**b**, **c**) in two different cases with COVID-19 pneumonia, showing the typical appearance of bilateral peripheral patchy ground-glass densities (**a**, **c**) and/or consolidations (**b**), subpleural band (black arrows), and vascular dilatation (blue arrows)

According to the CT findings, the most prominent features are GGO with vascular pleural thickening and interlobular septations, and parenchymal and subpleural bands were the most constant imaging features (Fig. 2).

A significant correlation was found between disease severity and consolidation, interlobular septation, bronchial thickening/dilatation, pulmonary nodule, and lymphadenopathy.

**Fig. 3:**
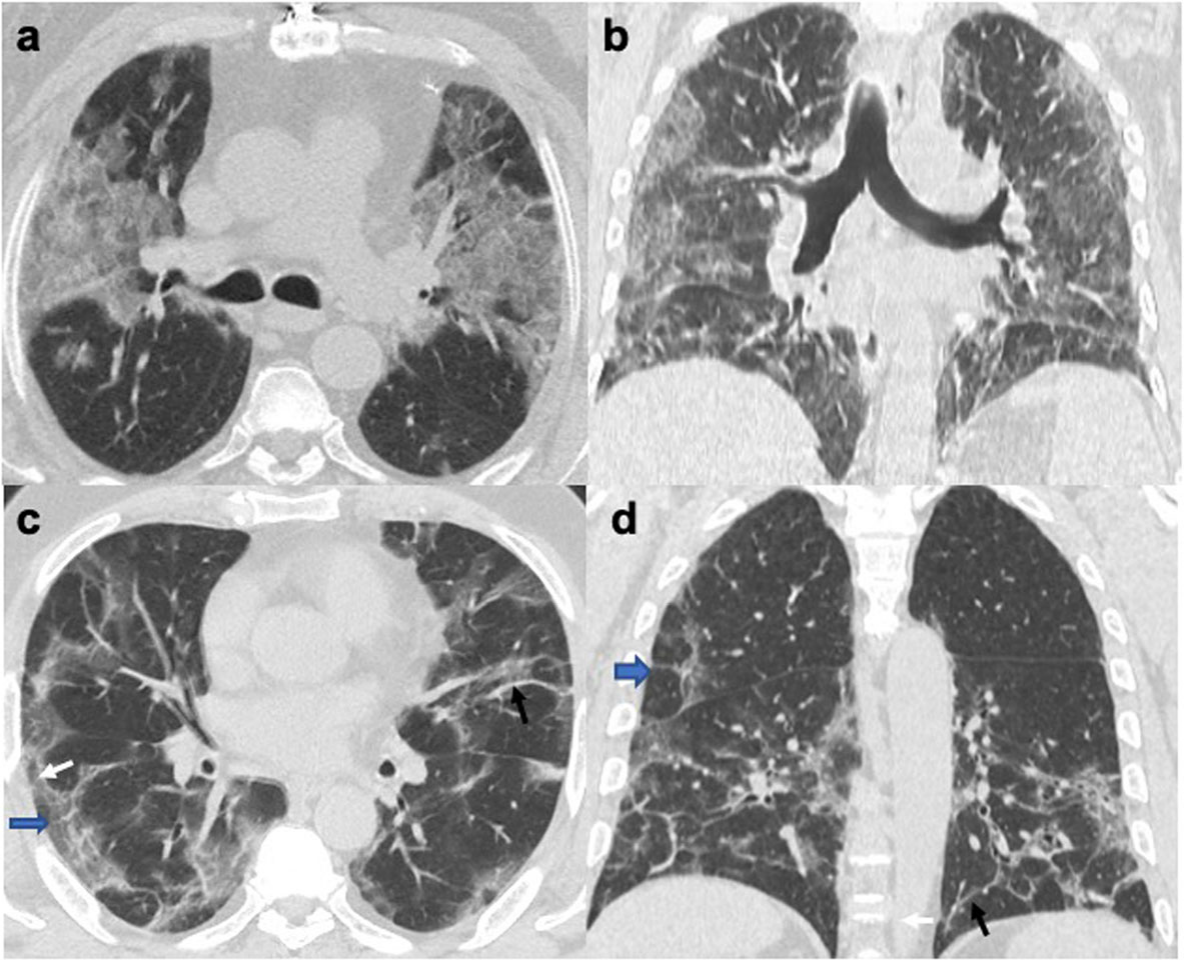
Axial (**a**, **c**) and coronal (**b**, **d**) CT slices of the lung window in different cases with COVID-19 pneumonia (**a**, **b**) showing the “crazy-paving sign” shown as bilateral ground-glass density with prominent interlobular and intralobular septations. **c**, **d** The bilateral parenchymal bands (black arrows), inter- and intralobular septations (blue arrows), and subpleural bands (white arrows), forming the spiderweb sign

**Fig. 4:**
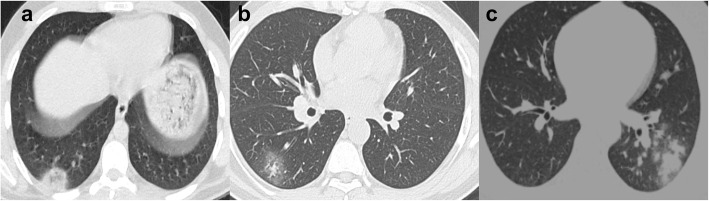
Axial chest CT lung window in different cases with COVID-19 pneumonia showing **a** the reversed halo sign: right lower lobe posterior basal segment patchy consolidation with internal lucency. **b** Halo sign: patchy consolidation with a surrounding halo of ground-glass density at the right lower lobe apical segment (**b**) and at the left lower lobe (**c**)

**Fig. 5:**
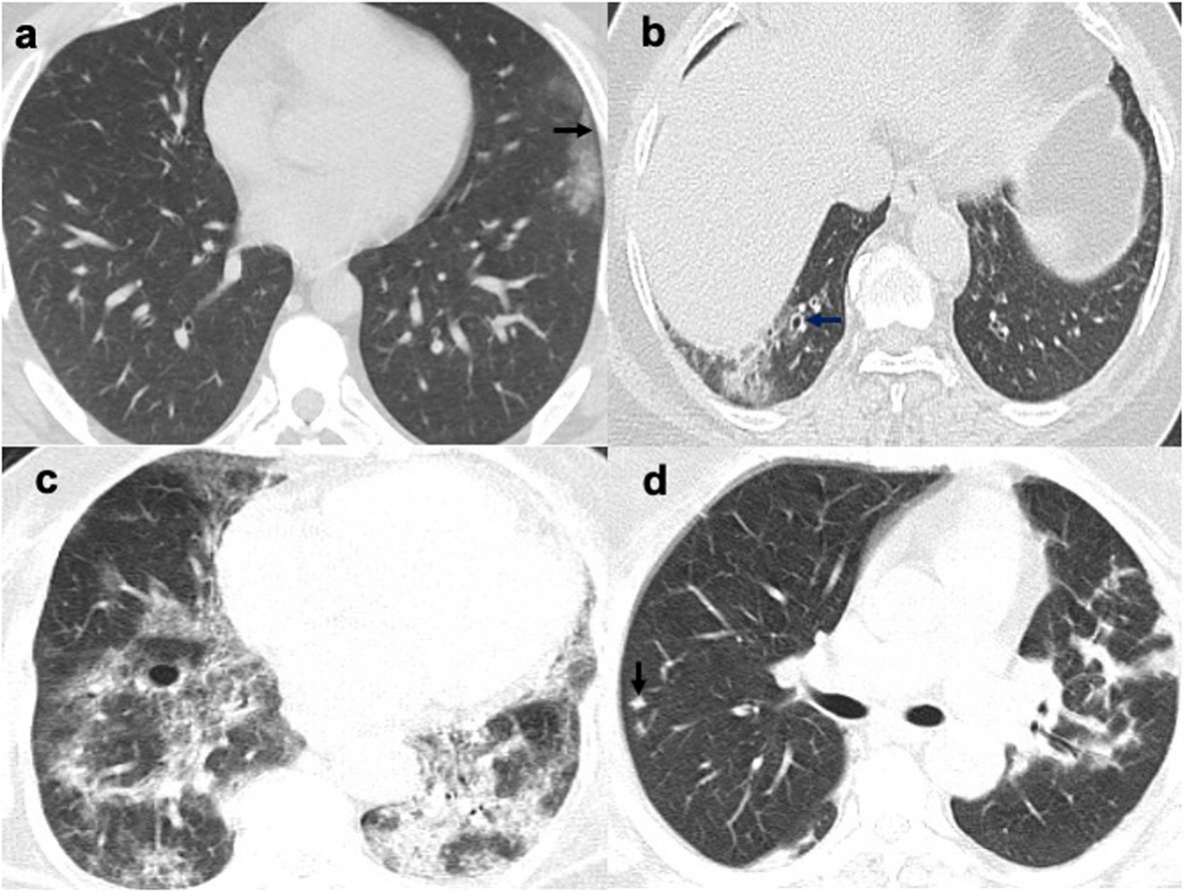
Axial chest CT lung window in cases with COVID-19 pneumonia. **a** The patchy ground-glass density with subpleural lucent line (arrow). **b** Right patchy ground-glass opacity, bronchial dilatation, and thickening (arrow). **c** The bilateral ground-glass densities and consolidation, with a right lower lobe cyst and a right apical segment LL angulated shape well-defined nodule (**d**)

Among several specific CT signs noted with COVID-19 pneumonia, the crazy-paving sign was the most encountered CT sign followed by the subpleural sparing sign. A significant correlation was found between disease severity and crazy-paving, halo, and reversed halo signs (Table 3 and Figs. 3, 4, and 5).

**Table 3:** CT chest findings in cases with COVID-19 pneumonia, number, and percentages of cases with a severity significance value

CT chest finding	Number and percentages of cases affected	Significance *p* value
Consolidation	60 (30%)	0.040*
Ground-glass opacity (GGO)	199 (99.5%)	0.738
Inter- and intralobular septations	144 (72%)	0.003*/0.110
Parenchymal bands	157 (78.5%)	0.456
Subpleural bands	138 (69%)	0.919
Bronchial thickening/dilatation	118 (59%)	0.003*
Vascular thickening	185 (92.5%)	0.179
Cyst	20 (10%)	0.116
Nodule	40 (20%)	0.005*
Pleural thickening	153 (76.5%)	0.133
Pleural reaction	6 (3%)	0.407
Pleural effusion	7 (3.5%)	0.369
Lymphadenopathy	66 (33%)	0.088*
Crazy-paving sign	73 (36.5%)	0.000*
Halo sign	22 (11%)	0.097*
Reversed halo sign	24 (12%)	0.082*
Spider web sign	23 (11.5%)	0.605
Subpleural sparing	53 (26.5%)	0.873

*p value < 0.1: significant

**Fig. 6:**
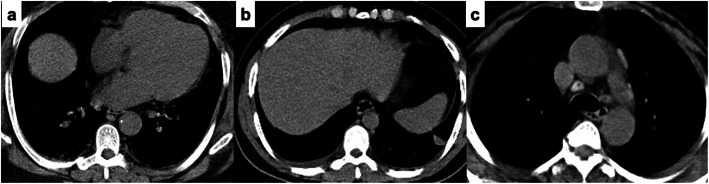
Axial CT mediastinal window in different cases with COVID-19 pneumonia. **a** Bilateral pleural thickening. **b** Right mild size free pleural effusion and left pleural reaction. **d** Mediastinal reactive lymph nodes noted at the right paratracheal, aortopulmonary, and prevascular regions. Note the high density of lymph nodes

Reactive lymphadenopathy exceeding 1 cm in short-axis diameter affected the paratracheal, retrocaval, and prevascular groups at levels 3A and 6 the most, followed by the level 5 lymph node group. The cortical estimated density ranged from 23 HU to 141 HU, with an average density of 91.8 HU (Fig. 6).

According to the CO-RADS classification, one patient was classified as CO-RADS 6 (known as COVID-19 pneumonia), 45 patients showed CO-RADS 4 findings, and 154 patients were classified as CO-RADS 5.

The CT-SS were from 1 to 24, with a mean value of 11.2 and a median value of 13. Among all patients, 40.5% had CT-SS of 11–15 (Table 4).

**Table 4:** Number and percent of cases in each severity score group

CT severity score	Number of cases
1–5	36 (18%)
6–10	47 (23.5%)
11–15	81 (40.5%)
16–20	31 (15.5%)
21–25	5 (2.5%)

## Discussion

COVID-19 is a highly infectious disease that has been spreading widely worldwide. Early diagnosis is an essential disease management strategy [11]. However, insufficient laboratory kits caused a challenge and dramatic dissemination of the disease [12]. Therefore, radiology, such as X-ray and CT, had become the principal method for diagnosis during the COVID-19 outbreak.

Chest CT could be an important complement for disease diagnosis as it assesses the extent and severity of the disease, which could express the disease burden [7]. Chest CT has a high sensitivity and a low specificity. Due to this low specificity, chest CT could hardly distinguish COVID-19 pneumonia from other diseases, such as community-acquired pneumonia and other non-infectious causes of acute GGO [3, 13].

The most common clinical symptoms of patients with COVID-19 are fever, cough, dyspnea, and fatigue [7]. In this study, fever and lower respiratory symptoms are the most common presenting symptoms in 69.5% and 66% of the patients, respectively.

The CT-SS was significantly higher in the > 75-year age group than in the 26–50-year age group (*p* = 0.0012). Furthermore, the CT-SS was significantly higher in the 51–75-year age group than in the 26–50-year age group (*p* = 0.0367). No statistical significance was observed in the CT-SS between the 51–75-year age group and the > 75-year age group (*p* = 0.3605). The CT-SS could help stratify patients’ risks and predict the short-term outcomes of patients with COVID-19 pneumonia [9]. Moreover, in this study, the 51–75-year age group had the highest CT-SS (*p* = 0.033).

Regarding disease distribution, all studies have indicated that COVID-19 has typical peripheral and subpleural distributions, and in most patients, COVID-19 involves multiple lobes, particularly the lower lobes [3]. In this study, 93.5% of all patients had bilateral involvement, whereas 89.5% had peripheral involvement. Higher severity scores for the lower lobe were observed in 55.5% of the patients. These results conform to those of the study by Salehi et al. [14] who reported bilateral involvement and peripheral distribution in 87.5% and 76.0% of their patients, respectively; however, many studies such as that by Zhou et al. [15] have reported that 77.4% of the patients had predominantly peripheral distribution of lesions; the mean CT-SS for the upper zone was significantly lower than that for the middle and lower zones, and no significant difference in the mean CT-SS was observed between the middle and lower zones [9].

In this study, 13 (6.5%) patients were unilateral with 11 cases (84.6%) involving the lower lobes, one involving both the upper and lower lobes, and one involving the middle lobe. Zhou et al. [15] have stated that in the early phase of the disease, the GGO may present as a unifocal lesion, most commonly located in the inferior lobe of the right lung.

Regarding CT chest findings, all studies have indicated that the main CT feature of COVID-19 pneumonia is the presence of multifocal bilateral patchy GGOs with or without consolidation and with interlobular septal and vascular thickening [3, 5]. In this study, the major CT abnormalities observed were GGO in 99.5%, vascular dilatation in 92.5%, parenchymal bands in 78.5%, interlobular septal thickening in 72%, subpleural band in 69%, and consolidation in 30% of the patients. These results conform to those of many studies that reported that the frequencies of different CT abnormalities were as follows: GGO was observed in 86–91% of the cases, consolidation in 39–63%, fibrotic streaks in 56.5%, subpleural line in 20–33.9%, and interlobular septal thickening in 59% [14–17]. However, Zhou et al. [15] have reported that 40.3% of the cases had GGO and 54.8% had microvascular dilation sign.

The characteristic sign was the “crazy-paving sign,” which was observed in 36.5% of the cases, which is characterized by the reticular interlobular septa thickening within the patchy GGO, which had been reported in SARS. The “spiderweb sign” was observed in 11.5% of the patients. The “spiderweb sign” is characterized by a triangular or angular GGO under the pleura with the internal interlobular septa thickened like a net. The adjacent pleurae were pulled and formed a spiderweb-like shape in the corner. Wu et al. [16] have reported that the frequency of the “crazy-paving sign” was 29% and that of the “spider web sign” was 25%. The reverse halo sign (atoll sign) (i.e., areas of GGO with peripheral consolidation) is frequently observed [3]; in this study, the frequency of the reverse halo sign was 12%. Furthermore, the halo sign, which is a consolidative nodule or mass with peripheral GGO, was found in 11% of the patients in this study; it is uncommon in adults and could reach 50% in children [18]. The pleural transparent line or subpleural sparing is another CT sign found in 26.5%, whereas in previous studies, the reported frequency of subpleural sparing was 6–53.2% [15, 16].

In this study, bronchial changes (bronchial thickening and distortion) were observed in 59% of the patients. Zhou et al. [15] have stated that 72.6% of the patients had air bronchogram, and 17.7% had bronchus distortion; however, Wu et al. [16] have reported that 11% of the patients had bronchial wall thickening.

In this study, 10% of the patients had air-containing cysts, which could be due to pathological dilatation or due to resorption of consolidation; some authors have described it as a cavity, and others called it cystic changes and cavity or bubble sign; this conforms to Shi et al.’s [12] study that found cysts in 10% of the cases. Nodules are characterized to be small (less than 3 cm) round, oval, or irregularly shaped and well or poorly defined opacity in the lung. It was reported in 3–13% of the COVID-19 CT cases, and most nodules are multifocal and irregular and can have a halo sign [19]. In this study, nodules were encountered in 20% of the patients.

In terms of pleural changes, in this study, pleural thickening was observed in 76.5% of the patients; however, pleural reaction and effusion were found in 1.5% and 3.5% of the patients, respectively. Zhou et al. [15] have shown that 48.4% of the patients had pleural thickening. However, pleural effusion was not significantly associated with COVID-19 pneumonia; its frequency ranged from 6 to 9% of the cases [15, 16, 20].

In this study, lymphadenopathy had a significant correlation with disease severity (*p* = 0.088). Lymphadenopathy occurred predominantly in patients with a severe form of the disease [21]. Mediastinal lymph node enlargement was found in 43.51% of the patients with COVID-19 pneumonia with hilar, and mediastinal lymph node enlargement was associated with a 2.79-fold increased risk of COVID-19 pneumonia [20]; however, Wu et al. [16] have reported that the frequency of mediastinal lymph node enlargement was 4% of the cases. In this study, lymphadenopathy was observed in 33% of the patients. The high density of lymph nodes without obvious calcification is not reported in the literature; therefore, it is suggested to be due to the association of CT evidence of old tuberculosis infection, which was noted in 12.5% of the cases.

The CT-SS of COVID-19 pneumonia has great significance in assessing the extent of pneumonia involvement, with differentiation of moderate, severe, and critical types, and in predicting the dynamic changes of chest CT follow-up exams in different severities of COVID-19 pneumonia. Furthermore, assessing the severity of COVID-19 in the early stage helps clinicians early and accurately treat the disease [22].

The CT-SS was proposed for the assessment of the extent of involvement in thin-section CT images. Zhou et al.’s scoring system divided both lungs into 12 zones altogether. The degree of involvement in each lung zone was scored from 0 to 4, with a maximum possible score of 48 [15]*.* In another scoring system, both lungs were divided into 20 regions, evaluated on chest CT using a system attributing scores of 0, 1, and 2; therefore, the sum of the individual may range from 0 to 40 points [7]. In this study, the lobar involvement scoring system (0–25) was used as it was practical and time-saving with such a high flow of cases [9, 10]*.*

Indicators of severe disease are marked tachypnea, hypoxemia, and infiltration of more than 50% of the lung fields [23]*.* According to Yang et al. [7], the optimal CT-SS threshold for identifying severe COVID-19 was 19.5/40, with 83.3% sensitivity and 94% specificity. In addition, Francone et al. [9] have stated that CT-SS of ≥ 18 is highly predictive of patient mortality in short-term follow-up. Therefore, in this study, 18/24 was considered the cutoff value among the mild and severe cases. The mean global CT-SS was 11.2 in this study. In Francone et al.’s study [9], the mean global CT-SS was 12.3 ± 11.1.

The main limitations of this study are that the number of severe cases was much lesser than that of mild cases, which might affect the statistical strength, and single doctor assessment and the disease duration were unknown.

## Conclusion

In Egyptian patients with COVID-19 pneumonia, multi-slice CT plays a vital role in early identification of the disease with the ground-glass densities, and peripheral adhesions were the most typical findings, Thus, radiologists should be familiar with other possible findings.

 The assessment of the CT-SS of COVID-19 is essential in determining the extent of pneumonia involvement, to help clinicians early diagnosis and accurately treat COVID-19 pneumonia. The mean CT-SS for Egyptian patients with COVID-19 pneumonia was 11.2 (mild severity).

## Data Availability

The datasets used and analyzed during the current study are available from the corresponding author on reasonable request.

## Abbreviations

COVID: Coronavirus disease
CT: Computed tomography
CT-SS: Computed tomography severity score
GGO: Ground-glass opacities
MSCT: Multi-slice computed tomography
SARS: Severe acute respiratory syndrome
